# A Unified Multi-Task Learning Model with Joint Reverse Optimization for Simultaneous Skin Lesion Segmentation and Diagnosis

**DOI:** 10.3390/bioengineering11111173

**Published:** 2024-11-20

**Authors:** Mohammed A. Al-masni, Abobakr Khalil Al-Shamiri, Dildar Hussain, Yeong Hyeon Gu

**Affiliations:** 1 Department of Artificial Intelligence and Data Science, College of AI Convergence, Sejong University, Seoul 05006, Republic of Korea; m.almasani@sejong.ac.kr (M.A.A.-m.); hussain.bangash@sejong.ac.kr (D.H.); 2 School of Computer Science, University of Southampton Malaysia, Iskandar Puteri 79100, Johor, Malaysia

**Keywords:** skin cancer, segmentation, classification, multi-task learning, joint reverse optimization

## Abstract

Classifying and segmenting skin cancer represent pivotal objectives for automated diagnostic systems that utilize dermoscopy images. However, these tasks present significant challenges due to the diverse shape variations of skin lesions and the inherently fuzzy nature of dermoscopy images, including low contrast and the presence of artifacts. Given the robust correlation between the classification of skin lesions and their segmentation, we propose that employing a combined learning method holds the promise of considerably enhancing the performance of both tasks. In this paper, we present a unified multi-task learning strategy that concurrently classifies abnormalities of skin lesions and allows for the joint segmentation of lesion boundaries. This approach integrates an optimization technique known as joint reverse learning, which fosters mutual enhancement through extracting shared features and limiting task dominance across the two tasks. The effectiveness of the proposed method was assessed using two publicly available datasets, ISIC 2016 and PH^2^, which included melanoma and benign skin cancers. In contrast to the single-task learning strategy, which solely focuses on either classification or segmentation, the experimental findings demonstrated that the proposed network improves the diagnostic capability of skin tumor screening and analysis. The proposed method achieves a significant segmentation performance on skin lesion boundaries, with Dice Similarity Coefficients (DSC) of 89.48% and 88.81% on the ISIC 2016 and PH^2^ datasets, respectively. Additionally, our multi-task learning approach enhances classification, increasing the F1 score from 78.26% (baseline ResNet50) to 82.07% on ISIC 2016 and from 82.38% to 85.50% on PH^2^. This work showcases its potential applicability across varied clinical scenarios.

## 1. Introduction

Skin cancer is one of the most commonly diagnosed malignancies in the world. Melanoma is a type of malignant skin tumor that develops when melanocyte cells expand out of control. Malignant melanoma is the most aggressive and deadliest type of skin tumor because it is more likely to invade adjacent tissues and has a higher mortality rate [1]. In dermatology, dermoscopy is commonly preferred over visual examination for assessing suspicious skin areas, as it provides a non-invasive, magnified (10–20×) view that enhances diagnostic accuracy. This imaging tool reveals deeper structures, such as vascular and hemorrhagic areas, aiding in the early and precise identification of skin abnormalities. In addition, dermatologists use diagnostic frameworks like the ABCDE rule [2,3] (Asymmetry, Border irregularity, Color variation, Diameter, and Evolution), which is critical for guiding melanoma detection.

Early detection and diagnosis of skin cancer is highly remediable and could reduce the mortality rate. However, the field of medical imaging faces challenges in accurately predicting diseases within the same organ due to the redundancy of surrounding tissue for different disease types (e.g., skin cancer). Due to the high visual similarity among various types of skin lesions, a health examination may result in an inaccurate diagnosis [1]. In practice, dermatologists still face difficulties in improving skin cancer diagnosis. This is due to the fact that manual assessment of dermoscopy images by experts is typically difficult, error-prone, time-consuming, and subjective (i.e., various diagnostic conclusions may result) [4,5]. As a result, the development of an automated and trustworthy computer-aided diagnosis (CAD) system for detecting and diagnosing skin cancer has become an important evaluation tool that offers dermatologists a second opinion, helping them make better judgments with better reliability. In the CAD system, the segmentation of skin tumor borders and the classification of skin malignancies are two crucial tasks. The lesions segmentation task is usually utilized to detect the precise location of the skin cancer, while the disease prediction task aims to distinguish the disease types. However, segmentation algorithms face challenges due to the complex nature of dermoscopy images, which exhibit large variations in size, shape, texture, and color. Additionally, artifacts such as hair, air bubbles, blood vessels, illumination issues, and ruler, sign, or ink markers further complicate the segmentation process.

Although the recent advancements in deep learning are attracting considerable attention in the field of skin lesion prediction, the current approaches still have limitations. For example, in a general approach proceeded as a single stage, the input dermoscopy images are passed directly into the classification methods such as convolutional neural networks (CNN), which may lead to less-than-optimal diagnosis [6,7]. As the decision-making process for cancer categorization relies solely on the regions of skin lesions, the inclusion of adjacent healthy tissues introduces redundant details and representations from various skin lesion types, leading to a negative impact on the diagnosis performance. To address this challenge, a prior lesion boundary segmentation stage is needed to improve abnormality recognition. Therefore, the most recent studies have been accomplished through two distinct stages that involve the segmentation of skin lesions and the classification of cancer types [5,8]. Despite that, such approaches still suffer from limitations. The learning of these two processes (segmentation and classification) is conducted independently, which could cause a loss of information due to the non-precise lesion segmentation. Also, there will be difficulties in integrating the two-stage approaches into a unified clinical process because they are implemented on disparate platforms. In recent investigations, there has been an exploration into the multi-task learning concept, in which two tasks are simultaneously learned within a single model [9,10]. However, such approaches are subject to certain limitations. In practice, training both tasks of distinct objectives without incorporating any regularization can lead to the dominance of one task over the other. This can result in a degradation of the overall performance of the model.

To address these problems, this work presents a multi-task deep learning method capable of jointly segmenting boundaries of skin lesions and differentiating between their anomalies. The proposed approach facilitates mutual enhancement between the tasks of segmentation and classification by developing a single multi-task learning strategy that effectively regulates and optimizes their shared and distinct features. The results show that the proposed approach has decreased inference time, reduced training duration, and enhanced predictive accuracy. The proposed multi-task CAD system demonstrates reliable results for skin lesion analysis and presents a second opinion that aids clinicians in their decision-making process. Furthermore, the proposed joint multi-task approach is expected to be feasible and applicable in clinical practices and can potentially be generalized to different domains.

The main contributions of this study are three-fold. Firstly, this paper introduces a unified multi-task learning method capable of jointly distinguishing between benign and malignant skin cancers and segmenting boundaries of skin lesions. Secondly, we propose a new optimization technique called joint reverse learning that facilitates mutual benefit across the two tasks by incorporating their common features and preventing one task from dominating the other. Thirdly, this study validates the feasibility and generalizability of our proposed joint multi-task learning method by testing it on an additional unseen dataset.

The goal of this research is to develop an integrated multi-task deep learning framework capable of accurately performing both skin lesion segmentation and classification within a single model, enhancing diagnostic precision and efficiency. Specifically, this study aims to overcome segmentation and classification limitations in traditional CAD systems through a joint reverse optimization technique and to validate the generalizability capability of the proposed multi-task approach.

## 2. Related Works

Following the enormous success of using machine learning to solve a variety of classification and clustering tasks in various fields, the past decade has witnessed many developments of artificial intelligence (AI)-based methods for the diagnosis of different medical diseases. We summarize the current skin lesion segmentation and diagnosis methods in the following subsections and in Table 1.

### 2.1. AI-Based Segmentation Methods

In recent years, AI-driven segmentation techniques [11,12,13] have demonstrated significant promise in assisting clinicians with the identification and diagnosis of skin tumors. For instance, fully convolutional networks (FCNs) were widely employed for the segmentation of skin lesions. For example, Yuan et al. [4] proposed a fully automatic framework based on deep CNN, which utilized the Jaccard distance-based loss function to mitigate the imbalance between the number of foreground and background pixels. Li et al. [14] introduce a dense model for skin lesion segmentation, which leverages residual learning, featuring dense deconvolutional layers, chained residual pooling, and hierarchical supervision. This architecture prioritizes dimension preservation, contextual feature fusion, and refined predictions through an end-to-end training approach, eliminating the need for prior knowledge or complex postprocessing. Al-masni et al. [15] proposed a full-resolution convolutional network that learned the full-resolution features of each pixel, leading to an improvement in the segmentation performance. Several researchers embedded the attention mechanism into a fully convolutional network to make the network focus on lesion areas. For instance, Xie et al. [16] proposed a CNN method configured to produce high-resolution feature maps that effectively preserve spatial details. The incorporation of spatial and channel-wise attention mechanisms in the model served to enhance representative features while mitigating the impact of noise. Similarly, Sun et al. [17] proposed a Multi-Scale Contextual Attention Network (MSCA-Net), which could learn the multi-scale contextual information for accurate skin lesion segmentation. Wu et al. [18] proposed an adaptive dual attention module that integrated global context modeling with multi-scale resolution fusion and spatial information weighting. The aim was to capture the continuity of lesion boundaries, reduce redundancies, and enhance segmentation performance. Other researchers studied skin lesion segmentation from different perspectives. For example, Cao et al. [19] introduced an approach that incorporates a pyramid transformer inter-pixel correlations module and a local neighborhood metric learning module within an encoder-decoder network. Likewise, the study presented in [20] introduced a contextual multi-scale network, which consistently integrates pyramid pooling and dilated convolution at each resolution level of the encoder. This research also explored the impact of incorporating test-time augmentation, named the inversion recovery scheme, during the evaluation phase. Wu et al. [21] proposed a feature adaptive transformer network, which captured long-range dependencies and global context information by employing an extra transformer encoder branch. An interesting work conducted by Tschandl et al. [22] demonstrated transferring encoder weights from a pre-trained network used for a classification task on images of the same domain to be further trained for a binary segmentation task. This study observed that the distinct sharing of the encoder may potentially contain useful information for segmentation.

In 2024, Zhu et al. [23] introduced a U-Net that integrated a Multilayer Perceptron (MLP), referred to as double-spatial-shift MLP, to enhance connectivity between different spatial locations for skin lesion segmentation. They appended an extra module to the top of the encoder, named lighter external attention, with the aim of expanding the local receptive field and capturing the boundary continuity of skin lesions. Li et al. [24] addressed the challenges of blurred boundaries and the substantial semantic gap between the decoder and encoder through the introduction of a dual aggregation transformer with dual attention. The dual aggregation module is developed to establish a connection between the local feature details of CNN and the long-range modeling ability of the transformer, aiming to reduce the loss of semantic information. Additionally, the spatial attention mechanism is employed to alleviate blurred boundaries by establishing pixel-level feature relations across transformer blocks. Another study [25] also utilized a U-Net-based transformer for enhancing lesion edges. This was accomplished by incorporating an edge detection operator into the difference convolution, a multi-scale local attention block, and a global transformer block. Recently, other approaches for skin lesion segmentation have emerged, employing semi-supervised learning with Generative Adversarial Networks (GAN) [26] and unsupervised learning using an uncertainty self-learning network [27]. The latter method involved generating Class Activation Maps (CAMs) as saliency maps, encompassing foreground (skin lesion), background (normal tissue), and regions of uncertainty.

### 2.2. AI-Based Classification Methods

Machine learning-based skin lesion classification methods require extensive feature extraction and selection to achieve robust performance. The selected features are used as inputs to machine learning models, such as the support vector machine (SVM) [28] and K-nearest neighbor (k-NN) [29], in order to classify skin diseases. Researchers have widely used machine learning methods for skin lesion classification. For example, Hameed et al. [30] employed two approaches for categorizing skin dermoscopy images. The first approach involved a three-category classification task, distinguishing between healthy skin and inflammatory and non-inflammatory diseases. The second approach expanded the classification to six categories, including healthy skin, acne, eczema, psoriasis, benign conditions, and malignant conditions. Various classifiers, namely decision tree (DT), SVM, k-NN, and ensemble classifiers with different kernels, were utilized for both classification tasks. The findings revealed that as the number of categories increased, the classification accuracy decreased for all classifiers. Interestingly, the quadratic SVM outperformed other classifiers, exhibiting the highest accuracy in both classification tasks. Furthermore, Hameed et al. [31] developed a multi-class multi-level (MCML) model for the classification of skin diseases into multiple categories. The MCML algorithm was implemented using two different approaches: the conventional machine learning approach and the deep learning approach. Notably, the MCML model demonstrated superior accuracy in classifying skin diseases. Xie et al. [32] proposed a network ensemble model for a skin lesion classification. The model integrated back propagation (BP) neural networks with fuzzy neural networks to achieve accurate classification performance. Abbes et al. [33] proposed a CAD system based on fuzzy decision ontology for melanoma detection in skin lesion images. After feature extraction, the system used fuzzy decision rules and the k-NN method to classify lesion images. Deep learning techniques have the capability to directly handle unprocessed image data, eliminating the requirement for a preliminary feature preparation phase. However, it is important to note that these methods came with increased computational costs [34]. For example, Ali et al. [35] proposed a deep convolutional neural network (DCNN) model for skin lesion classification. The model achieved better classification accuracy, with less computation time than transfer learning models like DenseNet, ResNet, AlexNet, VGG-16, and MobileNet. Patil and Bellary [36] proposed a non-invasive stage classification CNN model of melanoma skin cancer. The model utilized a similarity measure for text processing (SMTP) as a loss function. In a recent systematic literature review conducted by [37], several federated learning and transfer learning models for the classification of both melanoma and nonmelanoma skin cancers were explored.

### 2.3. Integrated AI-Based Methods

In [38], Mahbod et al. examined the effects of incorporating skin lesion segmentation masks on the performance of dermoscopy image diagnosis. They suggested that employing segmentation masks in a proper manner can substantially enhance the overall diagnosis performance of skin cancers. Numerous techniques have been suggested for simultaneously segmenting and classifying melanoma skin cancers, falling into two main groups: non-end-to-end and end-to-end approaches. In the former category, two distinct models are often trained separately for segmentation and classification, with their predictions serving as supplementary inputs for each other, facilitating the exchange of valuable information. Most non-end-to-end approaches concentrate on leveraging segmentation outcomes to improve classification performance [5,8]. For example, Dhivyaa et al. [39] used different methods for segmenting the skin lesion and then classified the lesion using decision trees and random forest algorithms. Balaji et al. [40] proposed a model for skin lesion classification, which used a graph cut algorithm for skin lesion segmentation followed by a Naïve Bayes classifier. Batista et al. [41] designed a model for skin lesion classification using deep and active learning techniques. They considered two segmentation strategies: The U-Net CNN model and the fully convolutional networks (FCN), which were manually corrected by the specialist. Gonzalez-Dıaz [42] introduced DermaKNet, a CAD system for automated skin lesion diagnosis. DermaKNet combined the expertise of dermatologists with a CNN-based framework, by incorporating specialized computational blocks to model discriminative properties of lesions. Kadirappa et al. [43] introduced an automated skin lesion analysis method, which demonstrated high accuracy in segmentation using the U-Net architecture with a Spatial Attention Block (SPAB) and achieved robust classification through the fusion of global and local features. Xie et al. [44] investigated the interdependence between skin lesion segmentation and classification tasks, introducing the mutual bootstrapping deep convolutional neural networks (MB-DCNN) model to address both tasks. In MB-DCNN, the coarse segmentation task generated an initial lesion mask, serving as prior knowledge to aid in classification. Concurrently, the localization of lesion maps from the classification network contributes to refining the segmentation.

Conversely, the second category of approaches, referred to as end-to-end, utilized a single model for simultaneous skin lesion classification and segmentation [45,46]. This is accomplished through the implementation of multi-task learning, where shared parameters for feature extraction are employed, and distinct cost functions are integrated [47]. Consequently, these methods facilitated the sharing of useful information across various learning tasks [9,10,48]. For example, Song et al. [49] proposed an end-to-end deep learning framework that can perform skin lesion detection, segmentation, and classification tasks simultaneously. Similarly, He et al. [50] presented multi-task learning (MTL-CNN) for simultaneously segmenting and classifying skin cancers. In this approach, an auxiliary task, edge prediction, is incorporated to enhance the model’s ability to learn robust skin lesion boundaries. Additionally, several lesion area extraction (LAE) sub-modules were utilized to eliminate background noise from classification features, leveraging segmentation predictions. Meanwhile, Song et al. [51] developed a strategy to enhance the diagnosis of skin lesions by reframing multi-task learning as a multi-objective optimization problem, separating objectives from a Pareto efficiency perspective.

An emerging paradigm in biomedical diagnostics is the use of microwave reflectometry imaging [52,53], which has recently gained attention for its integration with artificial intelligence techniques for in-vivo skin cancer detection [54,55,56]. This approach addresses the diagnostic limitations of dermoscopy imaging, particularly in cases where skin lesion types exhibit high visual similarity. Microwave reflectometry measures the dielectric properties of skin lesions across various frequencies and combines these data with image analysis for improved diagnostic accuracy. By adopting this technology, dermatologists can effectively detect early signs of skin cancer and provide better patient care. Exploring this innovative direction holds significant potential for future research.

**Table 1:** Summary of previous works in skin lesion analysis, categorized by task type: segmentation, classification, or integrated.

Reference	Task	Technique	Dataset	TNR	TPR	Accuracy	F1 Score	IOU	DSC
Yuan et al., 2017 [4]	AI-based Segmentation Methods	Deep FCN with Jaccard distance	ISIC 2016	96.7	90.4	95.3	-	83.6	90.3
Li et al., 2019 [14]		Dense deconvolutional network	ISIC 2016	96.0	95.1	95.9	-	87.0	93.1
			ISIC 2017	98.4	82.5	93.9		76.5	86.6
Al-masni et al., 2018 [15]		Deep full-resolution CNN	ISIC 2017	96.69	85.40	94.03	-	77.11	87.08
Xie et al., 2020 [16]		High-resolution CNN	ISIC 2016	96.4	87.0	93.8	-	85.8	91.8
			ISIC 2017	96.4	87.0	93.8		78.3	86.2
			PH^2^	94.2	96.3	94.9		85.7	91.9
Wu et al., 2021 [18]		CNN with adaptive dual attention module	ISIC 2017	96.28	90.61	95.70	-	82.55	89.69
			ISIC 2018	94.10	94.2	94.70		84.4	90.8
Cao et al., 2023 [19]		Global and local inter-pixel correlations learning network	ISIC 2018	92.9	94.1	94.4	-	83.9	90.3
Al-masni et al., 2021 [20]		Contextual multi-scale multi-level network	ISIC 2017	96.23	87.69	93.93	-	77.65	85.78
Wu et al., 2022 [21]		Dual encoder with CNNs and Transformer	ISIC 2016	96.02	92.59	96.04	-	-	91.59
			ISIC 2017	97.25	83.92	93.26	-	-	85.00
			ISIC 2018	96.99	91.00	95.78	-	-	89.03
			PH^2^	97.41	94.41	97.03	-	-	94.40
Zhu et al., 2024 [23]		Multi-spatial-shift MLP-based U-Net	ISIC 2017	98.28	91.31	-	-	-	92.08
			ISIC 2018	97.71	90.15	-	-	-	91.03
			PH^2^	97.97	96.50	-	-	-	96.40
Li et al., 2024 [27]		Uncertainty self-learning network	ISIC 2017	93.7	88.6	90.5	-	68.5	80.5
			ISIC 2018	87.8	90.9	88.5	-	68.3	80.8
			PH^2^	93.1	93.6	92.4	-	80.1	88.9
Cheong et al., 2021 [28]	AI-based Classification Methods	SVM with the radial basis function	ISIC 2016	98.49	96.68	97.58	-	-	-
Xie et al., 2017 [32]		Back propagation and fuzzy NN	Xanthous	93.75	95.00	94.17	-	-	-
			Caucasians	95.00	83.33	91.11	-	-	-
Abbes et al., 2021 [33]		KNN and Feature extraction	Collected Dataset	89.00	96.00	92.00	-	-	-
Patil and Bellary, 2022 [36]		CNN with similarity measure	UCO AYRNA	96.33	96.03	96.0	95.96	-	-
Yu et al., 2017 [5]	Integrated AI-based Methods (non-end-to-end approaches)	Very deep residual networks	ISIC 2016	94.1	50.7	85.5	-	-	-
Al-masni et al., 2020 [8]		Full-resolution convolutional network	ISIC 2016	71.40	81.80	81.79	82.59	-	-
			ISIC 2017	80.62	75.33	81.57	75.75	-	-
			ISIC 2018	87.16	81.00	89.28	81.28	-	-
Dhivyaa et al., 2020 [39]		Decision trees and random forest	ISIC 2017	99.0	87.7	97.3	-	-	-
Balaji et al., 2020 [40]		Dynamic graph cut and Naive Bayes classifier	ISIC 2017	70.1	91.7	72.7	-	-	-
Kadirappa et al., 2023 [43]		SASegNet and EfficientNet B1	ISIC 2017	97.3	95.6	95.60	95.4	-	-
			ISIC 2018	95.4	92.5	92.73	92.8	-	-
			ISIC 2019	97.7	92.4	91.73	92.5	-	-
			ISIC 2020	92.4	90.6	91.19	90.7	-	-
Xie et al., 2020 [44]		Mutual bootstrapping DCNN	ISIC 2017	93.0	78.6	90.4	-	-	-
			PH^2^	93.8	95.0	94.0	-	-	-
Al-masni and Al-Shamiri, 2023 [47]	Integrated AI-based Methods (end-to-end approaches)	nnU-Net and FC-NN	**Segmentation**						
			ISIC 2016	-	-	-	-	-	89.03
			**Classification**						
			ISIC 2016	89.47	80.47	-	79.94	-	-
Jin et al., 2021 [48]		Cascade knowledge diffusion	**Segmentation**						
			ISIC 2017	96.1	88.7	94.6	-	80.0	87.7
			ISIC 2018	90.4	96.7	93.4	-	79.4	87.7
			**Classification**						
			ISIC 2017	92.5	70.0	88.1	-	-	-
			ISIC 2018	97.6	80.2	96.3	-	-	-
Song et al., 2020 [49]		End-to-end multi-task deep learning	**Segmentation**						
			ISIC 2017	98.5	88.8	95.6	-	84.9	91.1
			**Classification**						
			ISIC 2016	72.3	99.6	89.1	-	-	-
			ISIC 2017	73.1	97.7	81.3	-	-	-
He et al., 2023 [50]		MTL-CNN	**Segmentation**						
			ISIC 2016	97.5	93.8	97.2	-	87.9	93.4
			ISIC 2017	98.3	88.6	95.5	-	81.5	88.7
			Xiangya-Clinic	97.8	93.1	96.9	-	86.4	92.4
			**Classification**						
			ISIC 2016	96.3	67.3	88.5	-	-	-
			ISIC 2017	93.0	76.8	90.7	-	-	-
			Xiangya-Clinic	86.6	97.0	95.9	-	-	-
Yang et al., 2017 [9]		Multi-task deep learning	**Segmentation**						
			ISIC 2017	98.5	84.9	95.6	-	76.0	84.6
			PH^2^	96.0	97.3	96.5	-	88.2	93.1
			**Classification**						
			ISIC 2017	92.5	67.8	85.0	-	-	-
			PH^2^	93.6	94.3	93.3	-	-	-
Chen et al., 2018 [10]		MTL with feature passing module	**Segmentation**						
			ISIC 2017	-	-	94.4	-	78.7	86.8
			**Classification**						
			ISIC 2017	-	-	80.1	-	-	-

## 3. Materials and Methods

### 3.1. Dataset

This study employs two well-known and publicly accessible dermoscopy datasets to evaluate the proposed joint multi-task segmentation and classification method. Specifically, the first dataset (https://challenge.isic-archive.com/landing/2016/, accessed on 20 May 2023), known as the International Skin Imaging Collaboration (ISIC 2016), was initially introduced during the “Skin Lesion Analysis toward Melanoma Detection” challenge at the 2016 International Symposium on Biomedical Imaging (ISBI) [57]. The ISIC 2016 dataset is composed of 8-bit RGB images of varying dimensions, ranging from 540 × 722 to 2848 × 4288 pixels. This dataset contains a total of 1279 dermoscopy images, with 900 images designated for training and a separate 379 images reserved for testing. These images contain pathological skin lesions, each of which is labeled with a specific disease classification, namely benign nevi or melanoma. The ISIC 2016 dataset also includes binary masks for segmentation that precisely outline the segmented tumors against the neighboring normal tissue. Expert dermatologists have meticulously labeled and annotated both skin diseases and segmented lesions.

**Figure 1:**
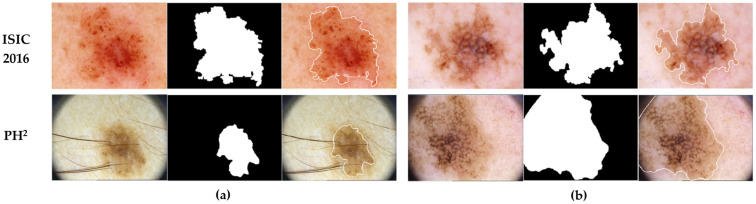
Paired instances of skin lesion dermoscopy images, along with their associated segmentation masks, sourced from the ISIC 2016 dataset and the PH2 dataset. (**a**) Shows benign cases, and (**b**) displays melanoma cases from both datasets. The third column in each case represents a visual display of the segmented lesion boundaries.

The second dataset (https://www.fc.up.pt/addi/ph2%20database.html, accessed on 15 January 2024), known as PH^2^, was obtained from the Dermatology Service of Hospital Pedro Hispano in Matosinhos, Portugal [58]. The PH2 dataset comprises 200 dermoscopy images in RGB format, consisting of 160 benign images (80 atypical nevi and 80 common nevi) and 40 melanoma images. All the images in this dataset have a consistent size of 768 × 560 pixels. These data also provide annotations for all segmented lesions. Notably, in this study, we employed the PH^2^ dataset as an additional unseen testing set to assess the feasibility and generalizability of our proposed multi-task learning approach. An overview of both dataset distributions and their respective data splits is presented in Table 2. Figure 1 illustrates examples of dermoscopy images from both the ISIC 2016 and PH2 datasets, along with their corresponding skin cancer classes, ground-truth segmentation masks, and segmented lesion boundaries.

### 3.2. Data Preparation

Proper data preparation is a crucial step in developing effective artificial intelligence models. It includes normalization, input image scaling, augmentation, and addressing the imbalanced classes. These steps allow the machine learning algorithms to learn from preprocessed data effectively, enhancing the overall quality of the model. In order to preserve consistency and foster stable convergence learning, we normalized each RGB dermoscopy image in a channel-wise manner, scaling them between zero and unity. Standardizing input size is a critical requirement for training and testing Convolutional Neural Network (CNN) models; therefore, we rescaled all images in ISIC 2016 and PH^2^ datasets to a fixed size of 192 × 256 pixels using bilinear interpolation. This process maintains a similar height-to-width aspect ratio and avoids any potential geometric distortion [4].

In order to effectively train deep learning networks, a larger number of training samples is required. To address this need, we applied various augmentation techniques to enlarge our training dataset. A total of 16 rotation and flipping transformations were applied to the original training images, including rotation with angles of 0°, 45°, 90°, 135°, 180°, 225°, 270°, and 315°, and four left-to-right and four up-to-down flipping operators. Notably, we observed an imbalanced distribution of data between the benign and melanoma classes. To address this imbalance, we employed a varied amount of augmentation operations for each skin cancer class. More particularly, we enlarged the benign images only four times using four rotations, while the melanoma images underwent augmentation 16 times. Table 2 shows the dataset sizes after applying these enlargement techniques. It is worth noting that these augmentations were only applied to the training dataset. 

### 3.3. What Is Multi-Task Learning (MTL)?

In the field of machine learning, multi-tasking is defined as the capacity to execute various learning tasks at the same time while exploiting commonalities and distinctions among them. In other words, multi-task learning refers to a single shared model that is capable of performing multiple tasks rather than individual models for each task. Multi-task learning offers several benefits, including, in theory, decreased inference time, enhanced predictive accuracy, increased data efficiency, and reduced training duration [59,60].

However, the quality of predictions may decrease when a network is required to make multiple predictions. This can result in reduced multi-task performance, where smaller independent networks may outperform a single shared network. This may be due to the dominance of one task over others, resulting in suboptimal performance [60]. Consequently, to overcome the aforementioned challenges and capitalize on the potential advantages of multiple learning tasks, it is essential to optimize the multi-task learning approach. For further details of multi-task learning methods and their applications to the medical imaging domain, refer to these review studies [61,62].

**Figure 2:**
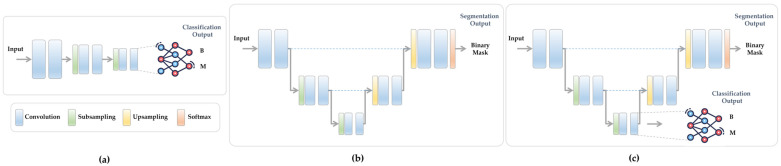
The concept of multi-task learning. (**a**) Pure classification model. (**b**) Pure segmentation model. (**c**) Multi-task learning model that combines both tasks in (**a**,**b**) with a shared encoder.

Figure 2 clarifies the concept of multi-task learning and how it integrates two individual models that perform different tasks of classification and segmentation (shown in Figure 2a,b) into a single shared model, as shown in Figure 2c. Even though the multi-task diagram illustrated in this Figure exhibits a shared encoder for both tasks, it poses the problem of one task potentially dominating the learning over the other. This simplistic strategy is referred to as basic multi-task learning. In the following sections, we demonstrate how our optimized multi-task learning methodology can overcome this shortcoming.

### 3.4. Proposed Multi-Task Learning Model

The proposed single multi-task deep learning model presents a computer-aided detection and diagnosis system that is capable of simultaneously segmenting the contours of skin tumors and differentiating between their abnormalities. Elaboration regarding the constituents of the proposed joint multi-task network is explained in the following subsections.

#### 3.4.1. Network Configuration

In this study, we adopt the architecture of the basic U-Net [63] as our backbone while employing the parameters and number of layers and features outlined in nnU-Net [64]. As is well known, the U-Net configuration is designed to achieve pixel-wise predictions since it contains two major pathways: encoder and decoder. An encoder pathway comprises convolutional and subsampling layers, which are responsible for extracting and learning contextual features of the input image with reduced receptive fields. Conversely, the decoder pathway includes upsampling and convolutional layers that refine the learned features, enabling the retrieval of dense predictions matching the size of the input image. In order to effectuate the multi-task learning model within the U-Net architecture, a straightforward approach involves connecting the learned features located at the last layer of the encoder pathway with extra dense Fully Connected Neural Networks (FCNN), thereby enabling predictions for other tasks, exemplified here by diagnosis outcomes (see Figure 2c). Consequently, the redesigned U-Net functions as a multi-task learning model with the capability to jointly handle the classification and segmentation tasks.

**Figure 3:**
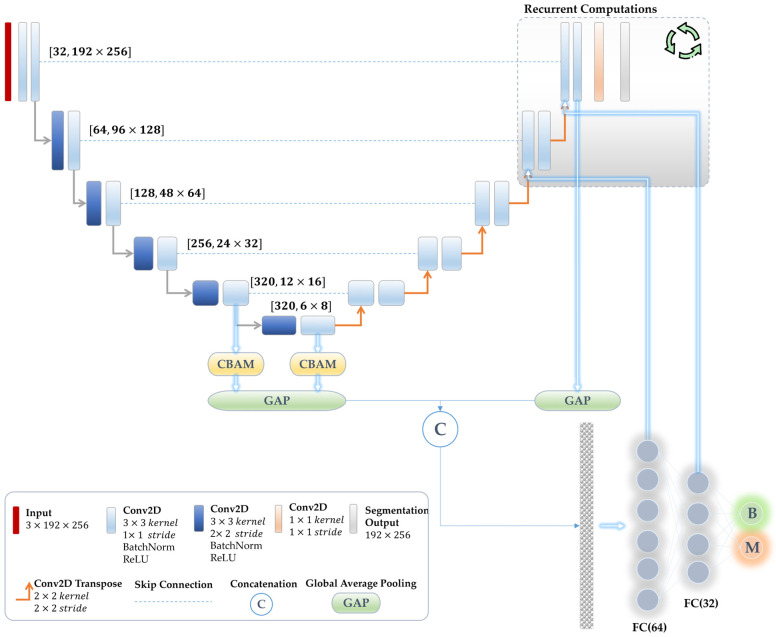
Scheme illustration of the proposed unified joint multi-task learning model for classifying skin abnormalities and segmenting their lesion boundaries.

In this study, we employ a U-Net architecture that consists of six convolution blocks with corresponding feature kernels of 32, 64, 128, 256, 320, and 320, respectively, which is similar to the nnU-Net framework. At each convolution block in the encoder, two subsequent convolution operations are applied with strides of 2 × 2 and 1 × 1 and filter sizes of 3 × 3, respectively. These convolutional layers are subsequently followed by batch normalization and Rectified Linear Unit (ReLU) activation. Here, we replace the subsampling operations in the encoder pathway with stride convolutions, fostering additional learning of pooling layers and enhancing the model’s overall stability [65]. An analogous configuration has been utilized in the decoder pathway, with the only difference being that all convolution operations have a stride of 1 × 1. Instead of utilizing the upsampling operation, we employ the transpose convolution. A convolutional layer with a kernel size of 1 × 1 and a stride of 1 × 1 is appended to the last layer of the encoder path, which employs a multinomial logistic regression referred to as the softmax classifier. Further elaboration of the network’s structure can be observed in Figure 3.

The proposed method employs a supervised learning strategy, enabling the model to learn and extract relevant features during training, thereby optimizing performance and minimizing computational loss. We input color RGB images into the network, allowing it to learn various features, which may include shading and irregular boundaries. The last layer in the segmentation path utilizes a softmax function to convert these learned features into a binary mask that delineates the suspicious region.

#### 3.4.2. Joint Reverse Learning

In practical implementation, simultaneously training tasks with distinct objectives and lacking any form of regularization may lead to one task overpowering the other (i.e., the dominance problem), ultimately causing a decline in diagnosis accuracy. Notably, our joint multi-task learning model seeks to promote mutual benefits between the diagnosis and segmentation processes. In particular, our focus is on developing a unified multi-task learning method that effectively regulates and optimizes the shared and distinct features of both tasks. This paper proposes three various solutions to address the above challenges and effectively manage the potential dominance of one task over the others.

Initially, our suggested joint multi-task learning method shares all the encoder layers for both segmentation and classification tasks, which is known as a parameter-sharing technique. Shared layers of the associated tasks diminish the likelihood of overfitting, allowing the network to learn representations that adeptly address both tasks and collaboratively enhance their performance. Second, we establish a linkage between the segmentation decoder output and the features of the classification path. This process serves as an attention mechanism involving the multiplication of the segmented regions with feature representations of the classification module. This leads to a focus on anomaly representations while ignoring normal and redundant features. Third, we develop a new optimization mechanism via reversely associating the weights in the FC-NN layers of the classification sub-module with the last resolution levels in the segmentation decoder pathway. The sequential nature of network implementation presents a challenge in executing this reverse connection. To overcome this, we conduct recurrent computations on parts of the network associated with this reverse connection. Specifically, the last two resolution levels of the decoder are computed twice: once for normal propagation to obtain the initial segmentation output, which is directly connected to the classification sub-module (the second solution scenario), and another computation based on the reverse connections from classification into segmentation. This reverse connection enables the updating of segmentation neural network weights based on prediction decisions, serving as further attention for segmentation improvement. These proposed solutions mitigate the issue of task dominance often encountered in multi-task learning. Figure 3 shows the detailed network structure featuring the aforementioned joint learning mechanisms.

Assume that $X_{L}^{k}$ represents a feature map at a particular convolutional layer L with k features. Then, the subsequent convolution layer can be computed as follows:

(1) $$X_{L}^{k}=\Phi \left(W_{L}^{k}\;*\;X_{L-1}^{k}+b_{L}^{k}\right),$$

where $W_{L}^{k}$ is the weights of convolutional kernels, $b_{L}^{k}$ is the bias applied to each layer, ‘∗’ is the convolution operator, and $\Phi \left(\cdot \right)$ is the ReLU activation function. Then, the reverse joint connection from the classification sub-module to the segmentation decoder layers can be expressed mathematically as follows:

(2) $$X_{L}^{k}=\Phi \left(W_{L}^{k}*\left[X_{L-1}^{k}\cdot {FC}_{cls}^{k}\right]+b_{L}^{k}\right),$$

where ${FC}_{cls}^{k}$ indicates the weights associated with the classification pathway, which corresponds to the same number of features k. Here, ‘·’ refers to the dot product operation that reflects the attention mechanism.

#### 3.4.3. Attention Mechanism

In addition to the joint learning strategies mentioned above, we demonstrate that integrating attention modules into the encoder path layers can lead to further improvement in classification performance. In this study, we employ a Convolution Block Attention Module (CBAM) [66] in the last two encoder resolution layers and directly attach their outputs to the classification sub-module, as presented in Figure 3.

**Figure 4:**
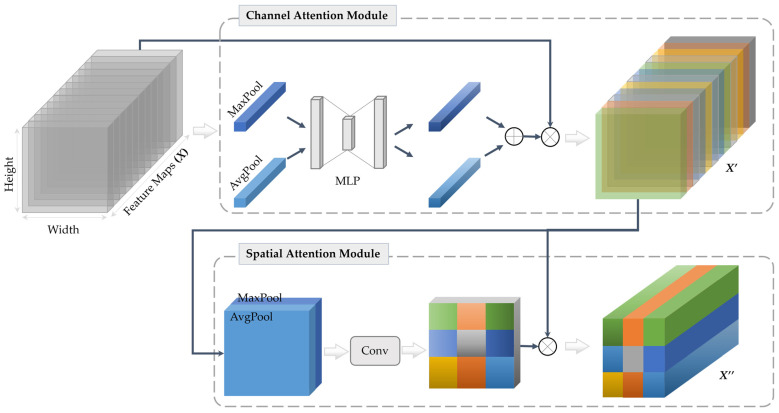
Diagram of the CBAM that integrates two sequential sub-attention modules, namely channel and spatial attention modules [59]. MLP refers to the multi-layer perceptron and $X^{\prime }$ and X″ represent the resulting feature maps by channel and spatial attention, respectively.

The CBAM integrates two sequential attention maps, namely the spatial and channel attention modules. The channel attention module bears a resemblance to the squeeze-and-excitation attention mechanism [67]. It facilitates the declaration of inter-channel relationships among processed feature maps and identifies the most significant map (i.e., feature detector). In contrast, the spatial attention module leverages the inter-spatial relationship of the feature maps to emphasize the most informative part (i.e., ‘where’ it is located). To provide further clarification on the CBAM, a plot [68] has been illustrated in Figure 4.

#### 3.4.4. Implementation Details

Our joint multi-task learning network utilizes a supervised learning approach, wherein labeled data are leveraged to optimize the network training process. Given that this work involves the training of both classification and segmentation tasks at the same time, we have employed separate loss functions in order to ensure effective training and convergence. For the classification and segmentation tasks, we employ the binary cross-entropy loss ($L_{BCE}$) and the Dice loss function ($L_{Dice}$), respectively. In order to efficiently optimize the multi-task issue, we maintain a balance in individual losses designed for each task [69]. To achieve this, a loss weighting technique that takes the control factor λ into consideration is used. When $\lambda_{1}>\lambda_{2}$, the classification task experiences adverse effects, while $\lambda_{1}<\lambda_{2}$ negatively impacts the segmentation task. Empirical experiments have led us to set $\lambda_{1}=\lambda_{2}=0.5$. The cumulative loss of this network is calculated using the formula below.

(3) $$L_{Total}=\lambda_{1}\cdot L_{Dice}+\lambda_{2}\cdot L_{BCE}$$

The Adam optimizer is utilized to optimize all hyper-parameters, with a batch size of 10 and an initial learning rate set to 0.003. The learning rate is then exponentially reduced by a factor of 10 during the training process. Network training reaches convergence at approximately the 50th epoch. The system implementation of this work was conducted using the Python programming language with the Keras and Tensorflow libraries on a PC equipped with a Cuda-enabled NVIDIA GeForce RTX 3080 GPU and 64 GB RAM.

### 3.5. Evaluation Measures

We quantitatively evaluate the proposed joint multi-task learning method using various measures, including true positive rate (TPR), also known as sensitivity, true negative rate (TNR), known as specificity, and F1 score for the classification task. Due to the image-level nature of this task, there is an imbalance between benign and malignant data in the test set. Therefore, we have adapted weighted measurements to address this issue. We also rely on the F1 score, which balances precision and recall and is especially suitable for imbalanced datasets. Moreover, we have utilized the confusion matrix to present a detailed distribution of the network predictions. In addition, we have employed distance measures, including the Dice similarity coefficient (DSC), the Mathew correlation coefficient (MCC), and intersection-over-union (IOU), also known as the Jaccard index, for the segmentation task.

## 4. Experimental Results

We evaluated this study using the original test set of the ISIC 2016 dataset, which comprises 379 images, including 75 and 304 melanoma and benign instances, respectively.

### 4.1. Baseline Experiments

In this section, we performed baseline experiments by implementing widely used deep learning methods: ResNet50 [70] for the classification task and U-Net [63] for the segmentation task. It is worth noting that both tasks were trained separately using the same training and testing sets. The achieved weighted F1 score for the classification model was 78.28%, while the segmentation model produced a Dice score of 91.04%. Based on these outcomes, we aimed to enhance the classification performance by incorporating the segmentation knowledge through the proposed multi-task learning strategy.

### 4.2. Ablation Study

The main objective of this section is to conduct experimental investigations of various ablation studies, with the aim of reinforcing the proposed multi-task learning method for simultaneous classifying and segmenting skin lesions in dermoscopy images. The base multi-task learning model that reflects the design architecture in Figure 2c is denoted as ‘MTL0’. In this experiment, no joint optimization was applied, and the features at the last encoder layer were passed to the FC layers to accomplish the classification task, while no changes were made to the decoder pathway. As a result of the absence of optimization in this base experiment, MTL0 achieved a segmentation Dice score of 90.03% and an overall prediction F1 value of 76.52%. These results revealed a decline in the segmentation and classification performances compared to the individual baseline experiments of U-Net and ResNet50 outlined in Table 3. This emphasizes how crucial it is to include regularization in the multi-task learning strategy to avoid one task overpowering the other.

The goal of the MTL1 experiment was to promote mutual benefit between both segmentation and classification tasks by merging representations from the last two blocks in the decoder with the features extracted from the last layer at the encoder in MTL0, as illustrated in Figure 3. MTL1, therefore, represented an optimization approach utilizing patterns from the segmentation decoder to enhance the classification task. More specifically, it incorporated additional lesion patterns into the FCNN classification layers while ignoring the surrounding healthy features, leading to an enhancement of the melanoma classification, achieving an F1 score of 77.96%. Nevertheless, the segmentation of lesion borders was adversely affected by this method, leading to a 1.17% reduction in DSC.

In the next MTL2 experiment, we applied joint learning that reversely connected the weights of the FC layers in the classification sub-module to the feature maps of the last two segmentation decoder layers. This recurrent computation mechanism allowed the model to focus on the segmentation path based on the classification predictions. Additionally, in this experiment, the last two encoder layers were fused with the classification sub-module. As reported in Table 3, the results showed that MTL2 effectively optimized shared patterns of both tasks, leading to an enhancement in the predicted F1 score performance from 77.96% to 80.66%, surpassing the performance of the baseline ResNet50 model. The results also showed that MTL2 enhanced the lesion boundary segmentation DSC performance from 88.86% to 89.15%.

For further improvement, we incorporated the CBAM attention module into the features obtained from the last two encoder layers in the third experiment (MTL3). Note that this experiment also leveraged the advantages of the preceding study (MTL2). Our results exhibited improvements in both tasks, achieving an F1 index of 81.00% for classification and a DSC of 89.37% for segmentation.

In the last experiment (MTL4), we sought to address the issue of class imbalance between two categories since the confusion matrices shown in Table 3 present decline predictions of melanoma images compared to the benign cases. The relatively lower accuracy of melanoma predictions compared to benign cases is due to the high visual similarity between the two classes. As presented in Section 3.2, the augmentation process alone was insufficient in addressing this issue, even with a high augmentation factor of 16 for melanoma cases. Thus, we increased the training data with an additional 404 melanoma images from the validation and training sets of ISIC 2017 to achieve a more actual balanced dataset. Importantly, we used the same testing dataset for evaluation. Our results demonstrated improvements in both tasks, with a diagnosis F1 value of 82.07% and a segmentation Dice score of 89.48%. Table 3 presents detailed results of all these experiments.

**Figure 5:**
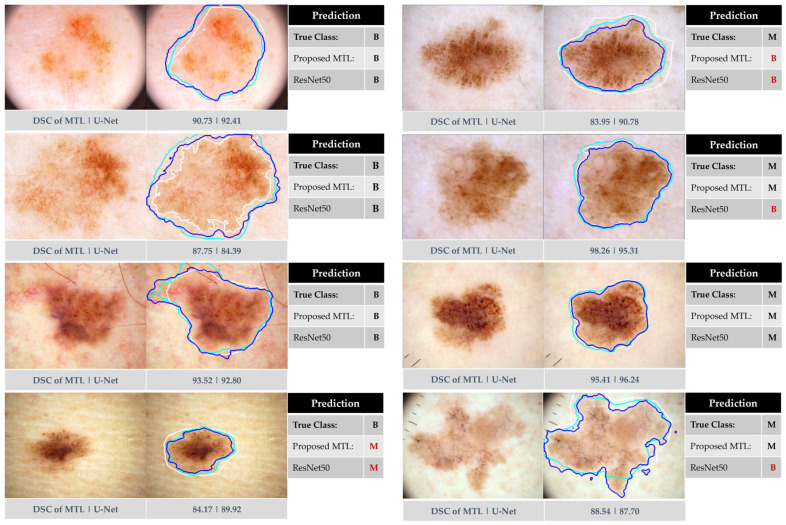
Exemplary results from our proposed approach using the ISIC 2016 test dataset in comparison to ResNet50 and U-Net. The left side corresponds to benign instances, while the right side indicates melanoma skin lesions. The segmented boundaries in blue, cyan, and white represent our proposed joint MTL approach, U-Net, and ground-truth mask, respectively.

**Figure 6:**
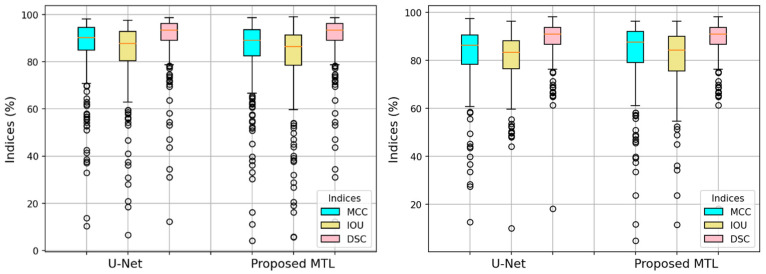
Boxplots of the segmentation performances per each test dermoscopy image in the ISIC 2016 test set (**left**) and PH^2^ dataset (**right**) in terms of MCC, IOU, and DSC.

Figure 5 displays exemplary results of our joint MTL approach in comparison to baseline networks (i.e., ResNet50 and U-Net). The Figure illustrates the anticipated dual outcomes of our approach: predicting dermoscopy images as either benign or melanoma skin cancer and precisely segmenting lesion borders. Figure 6 highlights this observation by presenting a boxplot that displays all segmentation measures for each individual test image. The results showed a remarkable similarity between the proposed MTL method and the U-Net model.

## 5. Discussion

This study investigated a multi-task learning (MTL) approach for simultaneous skin lesion segmentation and cancer-type classification (benign or melanoma). Our proposed joint reverse learning method aimed to ensure balanced learning between these tasks and prevent the dominance of one over the other. This is achieved by optimizing network training and facilitating self-attention across both tasks. Compared to single-task models, our MTL model, equipped with an optimization method involving forward and backward connections, achieved promising results in both tasks. It effectively discriminated between skin lesion abnormalities and demonstrated reasonable segmentation performance. These findings suggest the potential of this approach to improve the accuracy and efficiency of skin lesion diagnosis.

Our results, as shown in Table 3, demonstrate significant improvement in skin cancer classification, achieving a weighted F1 score of 82.07% compared to the baseline ResNet50’s 78.26%. This improvement comes with remarkable efficiency, as our proposed method uses only 10.90 million training parameters compared to ResNet50’s 23.84 million. This suggests that our MTL approach effectively extracts and learns robust features from segmented tumors during multi-task training. While the DSC score for segmentation dropped slightly compared to the U-Net baseline (89.48% vs. 91.04%), the overall improvement in lesion analysis and diagnosis outweighs this trade-off.

Figure 5 demonstrates successful segmentation by both U-Net and our MTL network, with Dice scores exceeding 84% for most tumors. However, incorrectly segmented lesions (underestimated or overestimated) impacted metrics significantly compared to ground-truth masks. Notably, our MTL method outperformed ResNet50 in classification, particularly for melanomas (second and last rows). Importantly, both methods struggled with the high visual similarity between benign and melanoma cases, as shown in the last example for benign cases and the first example for melanoma cases in the same Figure, highlighting the inherent challenge of accurate classification in these scenarios.

While the proposed MTL method achieves a slightly lower quantitative segmentation score (89.48%) compared to U-Net (91.04%), it is crucial to consider the practical implications. Visual inspection of Figure 5 reveals that most segmented regions in our method fall within acceptable ranges, suggesting high fidelity to ground-truth labels. Both approaches successfully handle challenging lesions with low contrast or irregular boundaries. These qualitative observations highlight that the slight reduction in score does not necessarily translate to significantly worse segmentation in practice. Furthermore, Table 3, Figure 5 and Figure 6 demonstrate the effectiveness of the joint learning scheme. It facilitates mutual benefit between network sub-modules, effectively addressing dominance issues and leading to improvements in both segmentation and classification tasks.

### 5.1. Evaluation of Additional PH^2^ Dataset

To assess generalizability and real-world applicability, we evaluated our MTL method on the unseen PH^2^ dataset (200 dermoscopy images, 160 benign, 40 melanoma). Note that the PH^2^ dataset was exclusively used for testing purposes. In other words, none of the models (i.e., proposed MTL, U-Net, and ResNet50) were trained using this dataset. Compared to ResNet50, our method achieved a significantly higher weighted F1 score (85.50% vs. 82.38%), as presented in Table 4. Notably, it excelled in melanoma classification, correctly identifying 34 out of 40 cases compared to the ResNet50 model, which correctly classified only 16 melanoma images. These results demonstrate the practical potential of our approach for accurate skin cancer diagnosis, particularly for challenging melanoma cases.

**Figure 7:**
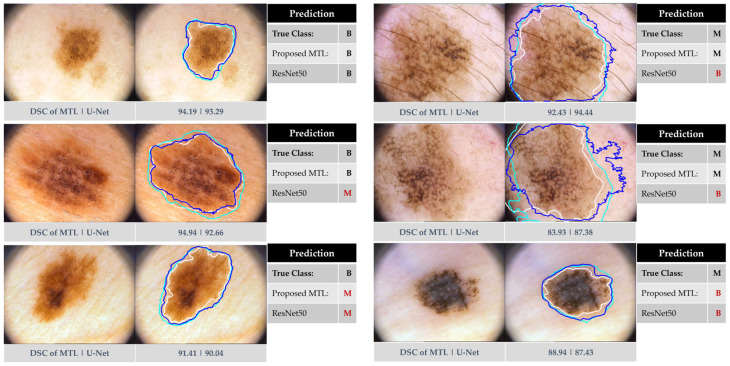
Exemplary results from our proposed approach using the PH2 dataset in comparison to ResNet50 and U-Net. The left side corresponds to benign instances, while the right side indicates melanoma skin lesions. The segmented boundaries in blue, cyan, and white represent our proposed joint MTL approach, U-Net, and ground-truth mask, respectively.

Figure 7 showcases segmentation and classification results from the PH^2^ dataset. While both methods generally perform well, our MTL approach exhibits superior classification, particularly for melanomas. Notably, both methods struggled with the visually similar cases in the last row, highlighting the inherent challenge of accurate classification in such scenarios.

Interestingly, on the PH^2^ dataset, our MTL method achieved slightly better segmentation performance (88.81% DSC) compared to the U-Net baseline (88.56%), unlike the ISIC 2016 results. This suggests that different datasets may respond differently to the MTL approach for segmentation. Despite the small improvement, Figure 7 visually demonstrates the effectiveness of our method in segmenting both benign and melanoma lesions on the PH^2^ dataset, as supported by the boxplot in Figure 6 (right). These findings highlight the potential of our method for generalizable segmentation, although further evaluation of diverse datasets is warranted.

### 5.2. Comparison Against Previous Works

We compared our MTL method against top entries from ISIC 2016 and other state-of-the-art approaches in Table 5. Focusing on melanoma classification performance measured by sensitivity (TPR) and specificity (TNR) for consistency, we found that our approach outperformed most single-stage methods. Distinguishing melanomas from benign lesions remained challenging in previous methods, with TPR scores ranging from 24.0% to 66.7%. Notably, our MTL method achieved a significantly higher TPR of 77.3%, representing a 6.5% improvement over the collaborative learning (CL-DCNN) model [71]. While MTL work in [47] achieved a higher TPR of 89.5%, its significantly lower TNR of 44.0% suggests potential overfitting or bias towards melanoma detection. Our TNR of 81.6%, while slightly lower than the best-performing method, indicates good generalizability to benign cases. These results demonstrate the effectiveness of our joint MTL approach in balancing sensitivity and specificity for melanoma recognition, offering promising potential for improved skin lesion analysis and medical image classification.

## 6. Conclusions

In this study, we demonstrated that utilizing a joint multi-task learning approach enhances the reliability of both skin tumor boundary segmentation and skin cancer diagnosis. The primary aim of this research was to suggest the feasibility of simultaneously performing different tasks using a unified multi-task learning approach. To achieve this goal, we introduced a method designed to regulate and optimize the network, facilitating effective learning for both segmentation and diagnosis. Our comparative analysis revealed that, although the segmentation performance of Multi-Task Learning (MTL) was slightly below that of U-Net on the ISIC 2016 dataset, significant improvements were observed in classification performance.

Despite these promising results, there is room for further advancement, particularly in improving overall diagnostic performance and model generalizability. Limitations of the current study include its focus on binary classification and the inherent imbalance within the datasets. Future work will aim to expand the model to encompass multiple skin cancer classes, providing a more comprehensive diagnostic tool. Additionally, we intend to explore fairness learning approaches to mitigate potential biases arising from sample size disparities and ensure a more equitable performance across all lesion types.

## Figures and Tables

**Table 2:** Distribution of ISIC 2016 and PH^2^ skin cancers datasets. ○ and × represent the inclusion and exclusion of the augmentation process.

Dataset	Augmentation	Training Set			Testing Set			Total
		B *	M *	Total	B	M	Total	
ISIC 2016	×	727	173	900	304	75	379	1279
ISIC 2016	○	2908	2768	5676	304	75	379	6055
PH^2^	×	−	−	−	160	40	200	200

* The abbreviations ‘B’ and ‘M’ denote the pathological categories of skin cancers, benign and melanoma, respectively.

**Table 3:** Experimental results of the proposed joint MTL model for diagnosis and segmentation of skin cancers, alongside baseline studies employing individual ResNet50 and U-Net methods.

ID	Experiment Details	Param. [M]	Classification Measurements						Segmentation Measurements		
				B	M	TPR	TNR	F1 score	MCC	IOU	DSC
**Cls**	Baseline Classification via ResNet50 [70]	23.84	**B**	261	43	78.10	85.86	78.26	-	-	-
				85.86%	14.14%						
			**M**	40	35						
				53.33%	46.67%						
**Seg**	Baseline Segmentation via U-Net [63]	10.71	**B**	-	-	-	-	-	87.06	84.62	91.04
				-	-						
			**M**	-	-						
				-	-						
**MTL0**	Multi-Task Learning (Base)	10.76	**B**	274	30	78.10	90.13	76.52	86.10	83.15	90.03
				90.13%	9.87%						
			**M**	53	22						
				70.67%	29.33						
**MTL1**	Integrating Segmentation Decoder Path Features into Classification Sub-Model	10.80	**B**	274	30	79.16	90.13	77.96	84.89	81.56	88.86
				90.13%	9.87%						
			**M**	49	26						
				65.33%	34.67%						
**MTL2**	Joint Reverse Learning from Classification to Segmentation	10.86	**B**	277	27	81.53	91.12	80.66	84.74	81.95	89.15
				91.12%	8.88%						
			**M**	43	32						
				57.33%	42.67%						
**MTL3**	CBAM Attention Module	10.90	**B**	277	27	81.79	91.12	81.79	85.07	82.08	89.37
				91.12%	8.88%						
			**M**	42	33						
				56.0%	44.0%						
**MTL4**	More Melanoma Data	10.90	**B**	248	56	80.74	81.58	82.07	85.46	82.46	89.48
				81.58%	18.42%						
			**M**	17	58						
				22.67%	77.33%						

**Table 4:** The performance of skin lesion segmentation and classification of the proposed multi-task learning approach were compared to U-Net and ResNet50 models using the PH2 dataset.

Method	Classification Measurements						Segmentation Measurements		
		B	M	TPR	TNR	F1 Score	MCC	IOU	DSC
Baseline Classification via ResNet50 [70]	**B**	152	8	84.0	95.0	82.38	-	-	-
		95.0%	5.0%						
	**M**	24	16						
		60.0%	40.0%						
Baseline Segmentation via U-Net [63]	**B**	-	-	-	-	-	82.10	80.44	88.56
		-	-						
	**M**	-	-						
		-	-						
Proposed Multi-Task Learning	**B**	135	25	84.50	84.38	85.50	82.27	81.0	88.81
		84.38%	15.63%						
	**M**	6	34						
		15.0%	85.0%						

**Table 5:** Performance comparison of our proposed MTL approach against other state-of-the-art methods and top-5 methods on the ISIC 2016 skin lesion classification dataset. The TPR and TNR are unweighted measures.

Method	Number of Stages	TPR (%)	TNR (%)
CUMED [5] (1st)	non-end-to-end two stages (learned independently)	50.7	94.1
GTDL (2nd)	single stage (VGG-19)	57.3	87.2
BF-TB (3rd)	single stage (N.A.)	32.0	96.1
ThrunLab (4th)	single stage (Inception v3)	66.7	81.6
Jordan Yap (5th)	single stage (N.A.)	24.0	99.3
ResNet50 [70]	single stage	46.7	85.9
GP-CNN-DTEL [72]	non-end-to-end two stages (learned independently)	32.0	**99.7**
MTL [47]	end-to-end two stages (joint learning)	89.5	44.0
MTL-CNN [50]	end-to-end two stages (joint learning)	67.3	96.3
Proposed MTL	end-to-end two stages (joint learning)	**77.3**	81.6

## Data Availability

The authors confirm that the data used in this study are publicly available at https://challenge.isic-archive.com/landing/2016/ & https://www.fc.up.pt/addi/ph2%20database.html (First dataset accessed on 20 May 2023. Second dataset accessed on 15 January 2024).
